# Online-Training für Hörimplantatchirurgie: Ein neuer Ansatz in der otologischen Ausbildung

**DOI:** 10.1007/s00106-024-01451-w

**Published:** 2024-03-26

**Authors:** Kristen Rak, Stefan Kaulitz, Johannes Voelker, Tassilo Müller-Graff, Jonas Engert, Björn Spahn, Stephan Hackenberg, Peter Grasso, Rudolf Hagen

**Affiliations:** 1 Klinik und Poliklinik für Hals‑, Nasen- und Ohrenkrankheiten, plastische und ästhetische Operationen, Universitätsklinikum Würzburg, Josef-Schneider-Straße 11, 97080 Würzburg, Deutschland; 2 MED-EL Elektromedizinische Geräte Gesellschaft m.b.H., Innsbruck, Österreich

**Keywords:** Otologische Eingriffe, Online-System, Felsenbein, Fragebogen, Simulationstraining, Otologic surgical procedures, Online system, Temporal bone, Questionnaire, Simulation training

## Abstract

**Ziel:**

Angehende Otologen stehen vor der Herausforderung, die komplexen mikrochirurgischen Techniken insbesondere im Zusammenhang mit Mittelohreingriffen und Cochleaimplantationen zu beherrschen. Traditionelle mikrochirurgische Ausbildungsmethoden beinhalten klassischerweise Präparationsübungen an Post-mortem-Präparaten und die begleitete Operation unter direkter Anleitung und Aufsicht. Dies setzt entsprechend voraus, Zugang zu Präparaten zu haben, und die Möglichkeit, von erfahrenen Chirurgen im eigenen Haus angeleitet zu werden. Da dies nicht ubiquitär umzusetzen ist, wurde ein Online-Trainingssystem für otologische Eingriffe entwickelt.

**Material und Methoden:**

Das System besteht aus einem künstlichen Modell des Schläfenbeins und den regulären chirurgischen Instrumenten und Übungsimplantaten. Zentraler Bestandteil ist eine hochauflösende Kamera, die mit einer Video-Streaming-Plattform verbunden ist und eine Online-Supervision der chirurgischen Schritte der Auszubildenden durch erfahrene Otochirurgen ermöglicht. Darüber hinaus wurde eine Pre-Learning-Plattform entwickelt, die die Anatomie des Felsenbeins, Information zum Instrumentarium sowie aufgezeichnete Vorlesungen und Lehrvideos umfasst, die es den Auszubildenden ermöglicht, ihr Wissen vor der praktischen Übung zu überprüfen und zu vertiefen.

**Ergebnisse:**

In den 3 bisher durchgeführten Kursen haben insgesamt 28 Teilnehmer mit unterschiedlicher chirurgischer Erfahrung an diesem Online-Fortbildungsprogramm für Otochirurgie teilgenommen. Mithilfe eines Fragebogens wurde die subjektive Bewertung des Programms durch die Teilnehmer abgefragt. So konnten auch Bereiche identifiziert werden, in denen eine Optimierung zu einer noch solideren Fortbildungserfahrung führen könnte.

**Schlussfolgerung:**

Das vorgestellte Programm einer otochirurgischen Online-Ausbildung kann dazu beitragen, bestimmte Basisgrundsätze im „Remote-Verfahren“ zu vermitteln. Damit wird diese Möglichkeit eines otochirurgischen Trainings auch Ärztinnen und Ärzten ermöglicht, die keinen unmittelbaren Zugang zu den klassischen Vor-Ort-Trainingseinheiten haben.

## Abstract (en)

**Objective:**

Education in microsurgery of the ear includes staged training to allow for mastering of the complex microsurgical procedures, particularly in the context of middle ear reconstruction and cochlear implantation. Traditional surgical training includes temporal bone preparations by cadaver dissection and supervised operating room practice. As these on-site trainings are limited, there is a need to broaden education facilities in an on-line format. Therefore, a first basic on-line training for otosurgery was developed.

**Materials and methods:**

The system consists of an artificial temporal bone model together with a set of basic surgical instruments and implant dummies. As an essential part of the training kit, a high-resolution camera set is included that allows for connection to a video streaming platform and enables remote supervision of the trainees’ surgical steps by experienced otological surgeons. In addition, a pre-learning platform covering temporal bone anatomy and instrumentation and pre-recorded lectures and instructional videos has been developed to allow trainees to review and reinforce their understanding before hands-on practice.

**Results:**

Over the three courses held to date, 28 participants with varying levels of prior surgical experience took part in this otological surgical training program. The immediate feedback of the participants was evaluated by means of a questionnaire. On this basis, the high value of the program became apparent and specific areas could by identified where further refinements could lead to an even more robust training experience.

**Conclusion:**

The presented program of an otosurgical online training allows for basal education in practical exercises on a remote system. In this way, trainees who have no direct access to on-site instruction facilities in ear surgery now have the chance to start their otosurgical training in an educational setting adapted to modern technologies.

Angehende Otochirurgen stehen vor der Herausforderung, die anspruchsvollen Operationstechniken der otologischen Chirurgie zu erlernen. Herkömmliche chirurgische Ausbildungsmethoden beinhalten im Regelfall Übungen an Felsenbeinpräparaten und das stufenweise Einführen in die jeweiligen Operationen bei den entsprechenden Eingriffen unter Kontrolle von erfahrenen Ohrchirurgen. Solche Ausbildungsmöglichkeiten sind eigentlich nur an dafür ausgestatteten Zentren möglich. In Anbetracht solcher Limitationen weltweit wäre ein einfach aufgebautes Online-Schulungssystems für die otologische Chirurgie wünschenswert.

## Konzept

Die Otologie, das medizinische Fachgebiet, das sich mit der Diagnose und Behandlung von Erkrankungen und speziell mit Funktionsstörungen des Ohrs befasst, nimmt einen wichtigen Platz in der rehabilitativen Medizin ein. Grundlage für eine individuell angepasste Behandlung solcher otologischer Krankheitsbilder ist nicht nur ein fundiertes Verständnis der Anatomie, sondern auch ein hohes Maß an praktischer Erfahrung [12]. Die klassischen chirurgischen Ausbildungsmethoden beinhalten Übungen an Felsenbeinpräparaten und das Operieren unter Aufsicht erfahrener Otochirurgen, was beides ressourcenintensiv und nur begrenzt zugänglich ist. [2, 3, 5, 10, 11]. In einer Zeit des schnellen technologischen Fortschritts haben sich Online-Plattformen als relevanter Vorteil für die Ausbildung von konservativ und chirurgisch tätigen Ärzten erwiesen [9].

Somit besteht aktuell ein dringender Bedarf für einen modernen, ressourcenschonenden Ansatz in der Ausbildung in der Otochirurgie: die Entwicklung eines Online-Schulungssystems für die otologische Chirurgie unter Verwendung eines tragbaren, mit dem Internet verbundenen Felsenbeinlabors. Diese innovative Plattform wurde speziell für die Mittelohr- und Hörimplantatchirurgie entwickelt, die heute zentrale Aspekte der chirurgischen Hörrehabilitation beinhaltet. Das Potenzial eines solchen Systems geht weit über dieses begrenzte Thema hinaus und ist auch Basis für die Ausweitung des chirurgischen Online-Trainings auf eine breite Palette anderer chirurgischer Verfahren.

Das Konzept des Online-Trainings basiert darauf, den Teilnehmern ein künstliches Felsenbein [7, 13] zusammen mit einer Auswahl einfacher chirurgischer Instrumente und verschiedenen Implantatmodellen für die Hörrehabilitation postalisch zuzusenden. Dies stellt einen wesentlichen Unterschied zu vielen bestehenden virtuellen Trainingssystemen dar, die ausschließlich auf simulierten Umgebungen basieren [1, 8, 14]. Durch den Zugang zu authentischen, greifbaren Elementen der otologischen Chirurgie bietet die Plattform eine praktische Anwendung, die in der derzeitigen ortsunabhängigen chirurgischen Ausbildung einzigartig ist. Im derzeitigen Konzeptstadium sind noch keine Bohrarbeiten, sondern nur haptische Betriebssimulationen geplant.

Im Zentrum der Methode steht die Echtzeitsupervision durch erfahrene Otochirurgen. Diese erfolgt über eine mit dem Work-Package zugesandte hochauflösende Kamera, die an eine Video-Streaming-Plattform angekoppelt werden kann, sodass die Tutoren vor Ort die chirurgischen Schritte der Auszubildenden „remote“ verfolgen können. Dieses ermöglicht eine unmittelbare und persönliche Beratung und Anleitung. Diese Integration der Betreuung in die Online-Ausbildung kann die Lücke zwischen der konventionellen chirurgischen Ausbildung „on-site“ und den Vorteilen des Online-Lernens schließen.

Dieser Online-Ausbildungsansatz hat das Potenzial, einer größeren Zahl von Fortbildungsinteressierten eine solide Basis für otochirurgische Kenntnisse zu ermöglichen. Entscheidender Vorteil ist die globale Verfügbarkeit, sodass der Erwerb chirurgischen Fachwissens nicht durch geografische Beschränkungen eingeschränkt wird [12].

## Materialien

- Audio-Video-Kommunikation,
- flexible exoskopische Makrokamera,
- künstliches Felsenbeinmodell,
- chirurgisches Instrumentenpaket,
- Plattform zur Vorbereitung auf das Lernen,
- voraufgezeichnete Vorlesungen und Lehrvideos,
- Fragebogen.

Zur Ermöglichung der Echtzeitkommunikation zwischen Auszubildenden und erfahrenen Otochirurgen wird eine gängige Online-Kommunikationssoftware verwendet. Diese Software ermöglicht eine nahtlose Audio-Video-Interaktion und stellt sicher, dass die Auszubildenden „remote“ Anleitungen erhalten, Fragen stellen und ihre chirurgischen Verfahren mit erfahrenen Mentoren besprechen können.

Ein zentraler Bestandteil des Programms ist eine hochauflösende 2‑D-Makrokamera (5,0 Megapixel, Auflösung 2594 × 1944 Pixel), die über eine USB-Schnittstelle einfach mit einer entsprechenden Hardware (PC, Laptop, Smartphone usw.) verbunden werden kann. Diese Kamera bietet eine detaillierte Nahaufnahme des chirurgischen Bereichs und ermöglicht den Auszubildenden eine genaue Beobachtung und präzise Durchführung der Op.-Schritte.

**Figure 1:**
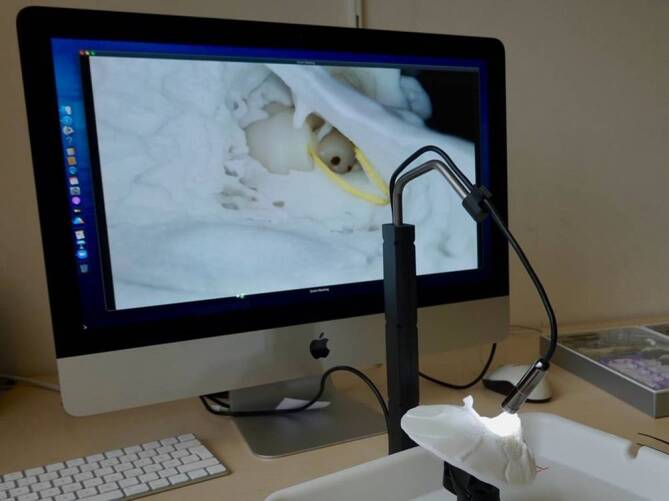

Das vorgebohrte künstliche Felsenbeinmodell verfügt über ein anatomisch korrekt nachgebildetes Mittelohr mit Gehörknöchelchen und Innenohrstrukturen. Ebenso sind die topographisch entscheidenden Strukturen – wie die Dura, der Sinus sigmoideus und der N. facialis mit Chorda tympani – als relevante Landmarken dargestellt. Um die Bedingungen von „echten“ Operationen zu simulieren, enthält das System ein Labyrinthmodell, das mit einem viskösen Medium gefüllt ist, welches die in der Cochlea vorhandenen Flüssigkeiten nachahmt. Dadurch können die Auszubildenden auch das Inserieren einer Cochleaimplantatelektrode durch das runde Fenster unter Bedingungen üben, die einer realen chirurgischen Umgebung sehr ähnlich sind (Abb. 1).

Um sicherzustellen, dass den Praktikanten alle notwendigen Instrumente zur Verfügung stehen, wird ihnen ein Paket mit den wichtigsten chirurgischen Instrumenten per Post zugesandt. Diese Instrumente wurden so ausgewählt, dass eine realitätsnahe Präparation im Felsenbeinmodell möglich ist.

Die Online-Plattform bietet ein umfassendes Vorbereitungsprogramm und umfasst Online-Vorlesungen zur Anatomie des Schläfenbeins und zur Instrumentierung. Den Auszubildenden wird erklärt, wie die Laborkomponenten einzurichten sind, damit sie vor Beginn der praktischen Ausbildung gut vorbereitet sind.

Um die Lernerfahrung weiter zu verbessern, bietet die „Pre-Learning-Plattform“ eine Sammlung von aufgezeichneten Vorträgen und Lehrvideos. Diese veranschaulichen jeden chirurgischen Schritt im Detail und ermöglichen es den Teilnehmern, ihr Verständnis vor der praktischen Übung zu festigen.

Den Teilnehmern wird als integraler Bestandteil des Schulungsprogramms ein Fragebogen übersandt. Dies ermöglicht das Sammeln und Auswerten von Rückmeldungen, sodass das Fortbildungsprogramm auf der Grundlage der Beiträge der Teilnehmer kontinuierlich angepasst werden kann. Dieser strukturierte Fragebogen spielt eine wichtige Rolle bei der Verbesserung der Gesamtqualität des Kurses und seiner Relevanz für die Bedürfnisse der otologischen Chirurgen und Auszubildenden.

So kombiniert das mobile Online-Trainings-Set alle erforderlichen Elemente, um eine ganzheitliche Lernerfahrung für otologische Chirurgen zu bieten. Die Auszubildenden werden mit den wesentlichen Materialien, Technologien und Bildungsressourcen ausgestattet, die sie benötigen, um die Eingriffe zu trainieren, und ebnen gleichzeitig den Weg für eine mögliche Ausweitung dieses Ansatzes auf weitere chirurgische Techniken.

## Methodik

### Ablauf des Kurses

Der vorgestellte Ansatz für die Ausbildung in der Otochirurgie basiert auf einem „Blended-Learning-Modell“. Blended Learning ist ein Bildungsansatz, der verschiedene Lehrmethoden gezielt kombiniert. Die Teilnehmer erhalten vorab per E‑Mail Vorbereitungsmaterialien, um sicherzustellen, dass sie die Kursinhalte vor Beginn des Kurses verinnerlicht haben. Es wird ein „Briefing“ organisiert, um sicherzustellen, dass die Teilnehmer die notwendigen Materialien über die digitale Plattform und per Post erhalten haben, dass ihre Einstellungen korrekt sind und dass sie auf den Kurs vorbereitet sind. Die Fortbildungsveranstaltung (Dauer 3 h) beginnt mit einer einführenden Live-Sitzung, in der der Tutor die Kursziele, die zu erwerbenden Fähigkeiten und die Bedeutung des Teilnehmerfeedbacks erläutert. Während des gesamten Kurses werden die chirurgischen Techniken anhand einer Kombination aus didaktisch sinnvoll aufbereiteten Videopräsentationen und Abbildungen vermittelt. Nach jeder Erklärung werden die Teilnehmer in Online-Gruppen eingeteilt, denen jeweils ein Tutor zur Seite steht, der in Echtzeit Anleitung und Unterstützung bietet. In diesen Gruppenräumen werden die Chirurgen nach Sprachen gruppiert, sodass die Teilnehmer nach Möglichkeit Unterstützung in ihrer Muttersprache erhalten und sich an Diskussionen beteiligen können. Dies fördert eine angenehmere und effektivere Lernumgebung, in der die Teilnehmer mit Gleichgesinnten interagieren können, die eine gemeinsame Sprache und einen gemeinsamen kulturellen Hintergrund haben. Das Hauptziel dieses neuartigen Fortbildungsansatzes besteht darin, das Selbstvertrauen der Teilnehmer zu stärken, indem sie praktische Erfahrungen im Umgang mit echten medizinischen Geräten und der Arbeit mit anatomischen Modellen sammeln, die der menschlichen Anatomie sehr ähnlich sind.

### Auswertung

**Figure 2:**
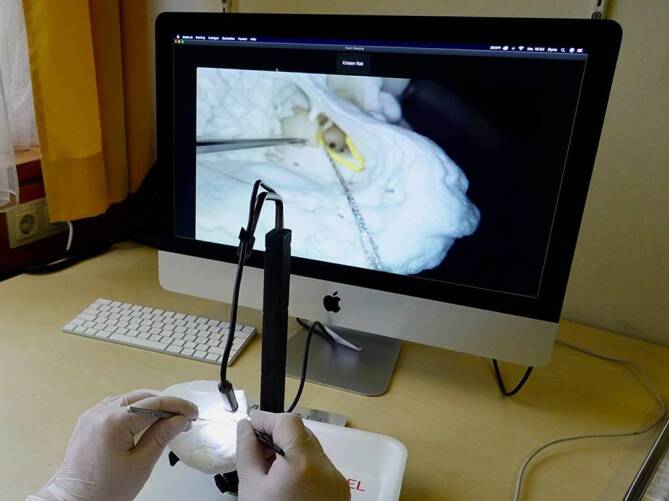

Im Anschluss an die Übungen wird den Teilnehmern über einen QR-Code ein Online-Fragebogen mit der Bitte um Beantwortung zur Verfügung gestellt. Die Auswertung ist freiwillig und anonym, sodass keine ethischen Bedenken hinsichtlich der Auswertung der Daten bestehen (Abb. 2).

## Ergebnisse

Das Schulungsprogramm hat sich als sehr effektiv erwiesen und erfreute sich internationaler Beteiligung. Bislang konnten Teilnehmer aus mehreren europäischen Ländern integriert werden, darunter Spanien, Deutschland und Österreich. Die Internationalität dieser Teilnehmer spiegelt die Fähigkeit des Programms wider, geografische Grenzen zu überwinden und Otochirurgen aus dem ganzen Kontinent Zugang zu solchen Fortbildungsmöglichkeiten zu verschaffen.

In den 3 bisher durchgeführten Kursen haben insgesamt 28 Teilnehmer an diesem otochirurgischen Fortbildungsprogramm teilgenommen. Diese hatten unterschiedliche chirurgische Vorkenntnisse, so z. B. Personen, die am Anfang ihrer otologischen Ausbildung stehen, aber auch solche, die ihre Fähigkeiten verfeinern wollten. Sie kamen aus verschiedenen medizinischen Einrichtungen und Praxisbereichen und trugen so zu einem reichhaltigen und kollaborativen Lernumfeld bei.

Diese heterogene Gruppe von 28 Teilnehmern hat gezeigt, dass das Programm ein breites Spektrum von Personen anspricht, die ein gemeinsames Interesse daran haben, ihre Kenntnisse und Fähigkeiten in der Ohrchirurgie zu erweitern. Der Erfolg des Programms bestätigt die Möglichkeiten, die Bedürfnisse eines breiten und internationalen Publikums abzudecken und letztendlich zur Entwicklung von otologischen Chirurgen in verschiedenen Regionen beizutragen. Die positiven Rückmeldungen und die internationale Beteiligung an diesen Online-Kursen zeigen das Potenzial für die weitere Entwicklung und Ausweitung dieses innovativen Ausbildungsansatzes.

**Figure 3:**
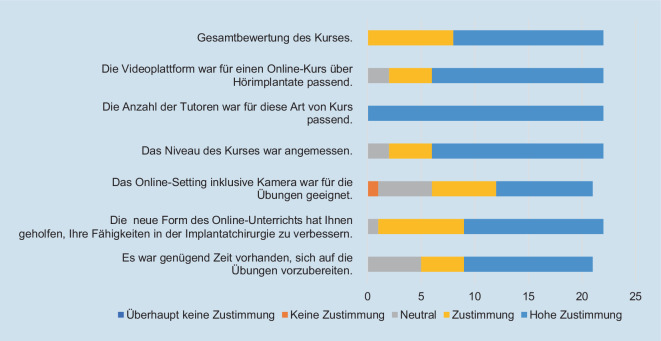

Der Kurs wurde mithilfe eines Fragebogens evaluiert. Den Fragebogen beantworteten 24 von 28 Teilnehmern. Die durchschnittliche Gesamtbewertung des Kurses betrug 1,36 (1 beste, 5 schlechteste). Dabei zeigten die eingegangenen Bewertungen zumeist eine starke Zustimmung oder Übereinstimmung mit dem Konzept, der Gestaltung und der Durchführung des Kurses (Abb. 3).

## Diskussion

Das positive Feedback der Teilnehmer zu dem neu etablierten Kurs unterstreicht das Potenzial eines solchen Programms. Obwohl dieser gut angenommen wurde, gibt es bestimmte Bereiche, in denen eine Optimierung zu einer noch solideren Ausbildungserfahrung führen kann.

Eine der größten Herausforderungen, die während der Schulung auftraten, war die 2‑D-Projektion der durch die flexible exoskopische Makrokamera aufgenommenen Bilder. Um diese Herausforderung zu überwinden, könnte der Wechsel zu einer 3‑D-Ansicht mit einem speziellen Display eine deutliche Verbesserung darstellen [6]. Eine solche 3‑D-Projektion könnte eine immersive und damit noch realitätsnähere Perspektive bieten, die der tatsächlichen chirurgischen Umgebung sehr nahekommen könnte. Dies würde auch die Betreuung durch die Tutoren und Ausbilder unterstützen. So würde die erhöhte Tiefenansicht präzisere Anleitungen ermöglichen, was bei den feinen und sehr komplexen anatomischen Strukturen von Vorteil wäre.

Ein weiterer Teilbereich, der verbessert werden kann, ist das für das Training verwendete künstliche vorgebohrte Felsenbeinmodell. Diese ermöglicht bisher kein haptisches Training des Bohrvorgangs, vereinfacht aber die Logistik und vermeidet die Erzeugung von potenziell schädlichem Staub beim Bohren [4]. Diese Hindernisse könnten überwunden werden, indem ein Weg gefunden wird, das Bohren ohne Staubentwicklung zu simulieren, oder indem zusätzliche Lernressourcen bereitgestellt werden, um die Teilnehmer mit dem Bohrprozess vertraut zu machen. So werden aktuell mehrere innovative Ideen evaluiert, um diese Herausforderung zu bewältigen.

Die beschriebenen Optimierungen haben das Potenzial, das Schulungsprogramm weiter zu verbessern und gleichzeitig die praktischen Probleme im Zusammenhang mit einem Bohr-Übungsprogramm in einem nichtklinischen Umfeld zu lösen. Da sich das Gebiet der otologischen Chirurgie ständig weiterentwickelt, wird die Anpassung des Schulungsprogramms mit 3‑D-Ansichten und verbesserter Simulation den Teilnehmern helfen, noch mehr Vertrauen in ihre chirurgischen Fähigkeiten zu entwickeln und diese zu beherrschen. Entscheidend für die Implementierung von technischen Optimierungen im Online-Felsenbein-Setup sind die einfache Umsetzbarkeit und Anwendung, damit das Programm für ein breites Spektrum von Teilnehmern zugänglich bleibt. Die technischen Hürden und Fehlerquellen müssen dabei möglichst niedrig gehalten werden.

## Fazit für die Praxis

- Die Entwicklung eines mobilen „Online-Felsenbeinkurses“ mit Online-Anbindung hat künftigen Otochirurgen einen neuen und damit innovativen Zugang zu einem qualitativ hochwertigen Training der Ohrchirurgie ermöglicht, der universal – also ortsunabhängig – eingesetzt werden kann und damit das allgemeine Niveau ohrchirurgischer Fähigkeiten weltweit verbessern kann.
- Der Einsatz von Blended-Learning-Techniken in Verbindung mit individuell gestaltbaren Online-Gruppenräumen ermöglicht ein dynamisches und interaktives Lernumfeld, in dem Teilnehmer mit unterschiedlichem Hintergrund und aus verschiedenen Ländern effektiv zusammenarbeiten können.
- Dieser Ansatz hat sich als erfolgreich erwiesen, wenn es darum geht, Kompetenz und Vertrauen bei den Teilnehmern aufzubauen.
- Die Rückmeldungen der Teilnehmer machten jedoch auch deutlich, dass weitere Verbesserungen notwendig sind, insbesondere um das taktile Erlebnis von Bohrübungen zu vermitteln.
- Um diese Herausforderung zu meistern, werden aktuell neue Ansätze geprüft, wobei der Schwerpunkt darauf liegt, den Aufbau auch weiterhin einfach und machbar zu halten.
- Zusammenfassend ermöglicht die Integration moderner digitaler Technologien in die praktische otochirurgische Ausbildung einen sinnvollen Einsatz solcher mittlerweile weit verbreiteter Medien für eine webbasierte Verbesserung allgemeiner otochirurgischer Grundkenntnisse, die auch den Patienten und der medizinischen Gemeinschaft insgesamt zugutekommen wird.
